# Multiresistance to antibiotics of Salmonella enterica serovar Typhimurium strains producing extended spectrum beta-lactamases (ESBLs)

**Published:** 2014

**Authors:** BI Coculescu, AM Palade, VL Purcarea

**Affiliations:** * “Titu Maiorescu” University, Faculty of Medicine, Bucharest; ** ”Cantacuzino” National Institute for Research and Development in Microbiology and Immunology, Bucharest; *** “Carol Davila” University of Medicine and Pharmacy, Faculty of Medicine, Bucharest

**Keywords:** Salmonella enterica serovar Typhimurium, ESBL (extended spectrum beta-lactamase), )multidrug-resistant strains (MDR)

## Abstract

The purpose of the study is to give the rising incidence of Salmonellosis and the continuous decrease of sensitivity of Salmonella to a range of antibiotics, the prime importance of the choice of optimal anti-infective chemotherapy in order to prevent the selection of multidrug-resistant strains (MDR). We have studied 54 strains of Salmonella sent for further investigation to “Cantacuzino” NIRDMI Bucharest. The statistical analysis was based on the interpretation pathogen susceptibility and analyzed according to CLSI recommendations 2009, which revealed that 8 (14,81%) identified as S. enterica serovar Typhimurium group, presented a phenomenon of MDR. S. Typhimurium strains producing ESBL showed resistance mechanisms associated with beta-lactam resistance to aminoglycosides, quinolones, sulfonamides and tetracyclines.

## Introduction

Gastrointestinal infections are caused by gram-negative amounts, ESBL-producing special problems in medical practice, by decreased sensitivity to antibiotics, due to the acquisition of various ways of achieving strength, including the production of ESBL [**1**]. Given that intestinal infections themselves are a public health problem, etiological involvement of microorganisms resistant to antibiotics increases their severity. A continuous monitorization of the emergence and spread of strains producing extended-spectrum beta-lactamases (ESBLs) is one of the major objectives of health programs that address infectious disease control.

## Materials and methods

We have studied 54 strains of Salmonella sent for further investigation to “Cantacuzino” NIRDMI Bucharest. Antibiotic sensitivity was tested by disc diffusion antibiogram method (Kirby-Bauer), by plating each inoculum on Mueller-Hinton medium according to CLSI recommendations (Clinical and Laboratory Standards Institute) by using microtablets supplied by Oxoid Ltd. (Basingstoke, UK) or Mast Diagnostics (Bootle, UK) by using an inoculum of 0,5 McFarland turbidity. E. coli ATCC 25922 and E. coli ATCC 35218 (for combinations of β-lactam beta-lactamase inhibitor) were used as control strains of pathogen susceptibility performed (ref. no. 0335P, MicroBioLogics) [**2**]. The following antibiotics were tested: Ampicillin (10 mg), Amoxicillin (10 mg), Amoxicillin/Clavulanic acid (20/10 mg), Cefoxitin (30 mg), Cefotaxime (30 mg), Ceftazidime (30 mg), Imipenem (10 mg), Nalidixic acid (30 mg), Ciprofloxacin (5 mg), Gentamicin (10 mg), Kanamycin (30 mg), Streptomycin (10 mg), Sulfonamide/Sulfadiazine (300 mg), Trimethoprim (5 mg), Cotrimoxazole (Trimethoprim 1,25/Sulfamethoxazole 23,75 mg), Tetracycline (30 mg), Chloramphenicol (30 mg) [**3**,**4**].

**Phenotype test for the confirmation of beta-lactam production - DDST (double disk synergy test) [**5**]**

The identification by producing extended-spectrum beta-lactamase (ESBL) was performed by using the double disc. The phenotypic confirmation of S. serovar Typhimurium strains producing suspected ESBLs was achieved by a simultaneous testing by Kirby-Bauer disc diffusion method, the synergy between ceftazidime discs, disc cefotaxime and amoxicillin with clavulanic acid. Oxoid discs containing a combination amoxicillin/clavulanic acid (20 μg/10 mg), ceftazidime (30 mg) and cefotaxime (30 mg) were used, placed at a distance of 2 cm (measured between centers disks) on the Mueller-Hinton medium. Plates were incubated for 18-20 hours at 37°C.

## Results and Discussion

Prevalence of Salmonella strains studied is the Enteritis serotypes (51,85%) and Typhimurium (27,78%) - see **Table 1**.

**Table 1:** Prevalence of enteropathogenic strains of Salmonella serovar group after characterization

Species	Group	Serovar	No strains	Source of isolation	(%)
S. enterica	D1	Enteritidis	28	21 ADD 7 FBD	51,85
S. enterica	B	Typhimurium	15	12 ADD 3 FBD	27,78
S. enterica	B	Agona	7	7 ADD (isolated)	12,96
S. enterica	B	Saintpaul	4	3 ADD (isolated) 1 FBD	7,41
ADD = Acute Diarrheal Disease FBD = Foodborne Disease					

The statistical analysis was based on the interpretation pathogen susceptibility and analyzed according to CLSI, revealing that of the 54 strains of Salmonella tested antibiotically, 8 (14,81%) identified as S. enterica serovar Typhimurium group, presenting the phenomenon of multiresistance to antibiotics (MDR), which meant being resistant to beta-lactams, aminoglycosides, quinolones, tetracycline and sulfonamides. High rates of resistance (>70%) were recorded to ampicillin, cefotaxime, ceftazidime, gentamicin, kanamycin and streptomycin, sulfonamide, trimethoprim, respectively nalidixic acid. The highest level of resistance to tetracycline was obtained (87,5%). Instead, the patient showed 100% sensitivity to cefoxitin, imipenem, and ciprofloxacin. Only 25% of the strains were sensitive to semi-synthetic penicillins and cephalosporins (3rd generation). In addition, a number of 7 strains showed resistance to chloramphenicol (87,5%). The same eight strains were resistant to amoxicillin + clavulanic acid, showing on one hand, the substrate specificity of beta-lactamases and, on the other hand, the possible adaptation of S. Typhimurium in clavulanic acid (a beta-lactamase inhibitor which remains active, the ESBLs, therefore will enhance the action of cephalosporins). According to the method of double diffusion synergy test, antimicrobial test results showed that the 8 strains tested produced beta-lactamase. Strains belonging to this phenotype (ESBLs) were characterized by cross-resistance to most beta-lactam tested. All the 8 strains showed ESBLs phenotype associated with phenotypes of resistance to aminoglycosides, cotrimoxazole and tetracycline, and of these, seven showed ESBLs phenotype associated with chloramphenicol resistance phenotype.

This indicative study on the characterization of antibiotic resistance by phenotypic methods strains of S. Typhimurium identified as producing beta-lactamases, showed that all the isolates were resistant to amoxicillin/clavulanic acid and ceftazidime. No strain sensitive to these antibiotics was identified. Carbapenems (imipenem) are beta-lactam antibiotics that were susceptible to all strains of S. Typhimurium producing ESBLs (100% of cases). Similarly, there was no resistant strain of isolated cefamicine (cefoxitim). The most active quinolone antibiotic ciprofloxacin proved that all the 8 strains of S. Typhimurium tested, showed sensitivity. All these antimicrobials may be used as antibiotics of choice in the gastrointestinal infections caused by Salmonella serovar Typhimurium.

In contrast, a high percentage of isolates showed resistance to tetracycline (87,5%), ampicillin, cefotaxime, gentamicin, kanamycin, streptomycin, sulfonamide, trimethoprim or nalidixic acid (the same percentage, 75%). In addition, the study phenotypes of acquired resistance to anti-infective chemotherapies revealed that the 100% strains (8 of 8 selected) of S. Typhimurium showed ESBL-producing multidrug-resistant phenomenon at medication (MDR) as diffusion antibiogram.

## Conclusions

Multiresistance to antibiotics of strains producing ESBLs was defined by the presence of concomitant resistance to aminoglycosides, fluoroquinolones and sulfamethoxazole-trimethoprim. S. Typhimurium strains producing ESBL showed resistance mechanisms associated with beta-lactam resistance to aminoglycosides, quinolones, sulfonamides and tetracyclines.

In this context, the increasing prevalence of Salmonella enterica serovar Typhimurium strains resistant to antibiotics and mainly those resistant betalactams, are worrying for the treatment of human salmonellosis.
